# Transanal drainage tube for the prevention of anastomotic leakage after rectal cancer surgery: a meta−analysis of randomized controlled trials

**DOI:** 10.3389/fonc.2023.1198549

**Published:** 2023-05-19

**Authors:** Shijun Xia, Wenjiang Wu, Lijuan Ma, Lidan Luo, Linchong Yu, Yue Li

**Affiliations:** ^1^ Shenzhen Hospital of Guangzhou University of Chinese Medicine, Futian, Shenzhen, China; ^2^ Shenzhen Traditional Chinese Medicine Anorectal Hospital, Futian, Shenzhen, China

**Keywords:** anastomotic leakage, transanal drainage tube, rectal cancer, anterior resection, meta-analysis

## Abstract

**Background:**

Anastomotic leakage (AL) is a serious complication of anterior resection for rectal cancer. The use of transanal drainage tubes (TDT) during surgery to prevent AL remains controversial. Therefore, we conducted a systematic review and meta-analysis of randomized controlled trials (RCTs) to determine the efficacy of TDT in reducing AL.

**Methods:**

Relevant data and studies published from inception until November 1, 2022, were retrieved from PubMed, Embase, and Cochrane Library databases to compare the incidence of AL after anterior resection for rectal cancer with and without TDT.

**Results:**

This meta-analysis included 5 RCTs comprising 1385 patients. The results showed that the intraoperative use of TDT could not reduce the incidence of AL after rectal cancer surgery (risk ratio [RR], 0.91; 95% confidence interval [CI], 0.52–1.59; p = 0.75). A subgroup analysis of different degrees of AL revealed that TDT did not reduce the incidence of postoperative grade B AL (RR, 1.18; 95% CI, 0.67–2.09; p = 0.56) but decreased the incidence of grade C AL (RR, 0.28; 95% CI: 0.12–0.64; p = 0.003). Further, TDT did not reduce the incidence of AL in patients with rectal cancer and a stoma (RR, 2.40; 95% CI, 1.01–5.71; p = 0.05).

**Conclusion:**

TDT were ineffective in reducing the overall incidence of AL, but they might be beneficial in reducing the incidence of grade C AL in patients who underwent anterior resection. However, additional multicenter RCTs with larger sample sizes based on unified control standards and TDT indications are warranted to validate these findings.

## Introduction

Anastomotic leakage (AL) is a serious complication of anterior resection for rectal cancer; it can prolong hospital stay, increase the economic burden of patients, and affect the postoperative incidence, mortality, and tumor survival rates (1, 2). Although significant advances have been made in surgical technologies and equipment, particularly in the use of laparoscopes and robots, the incidence of AL after rectal cancer surgery remains high at approximately 5%–19% (3).

Sex, age, diabetes, smoking, a history of radiotherapy and chemotherapy, intraoperative complications, anastomotic tension, distal site, and hypoperfusion have been identified as risk factors for AL (3–5). With regard to these risk factors, studies have reported various methods to reduce the incidence of AL. Currently, the construction of a diverting stoma in high-risk patients is a common preventive measure to relieve AL consequences (6–8). However, patients with a diverting stoma need to undergo a reoperation and incur increased treatment costs; moreover, the stoma may become permanent (9).

In the past decade, several studies have suggested that transanal drainage tube (TDT) can reduce the incidence of AL after anterior resection for rectal cancer (10–15). Some systematic reviews and meta-analyses support the preventive effect of TDT, but most of them are based on data with low research quality (16–18). Xiao et al. (19) reported that TDT effectively and safely reduced the incidence of symptomatic AL after anterior resection for rectal cancer. In contrast, a recent multicenter RCT by Zhao et al. (20) revealed that TDT did not play a role in preventing AL.

A meta-analysis of three RCTs showed that TDT were not effective in reducing the overall incidence of AL; however, they might be beneficial in reducing the incidence of grade C AL in patients who underwent anterior resection (21). However, considering the small number of studies included in this meta-analysis, the preventive effect of TDT cannot be adequately analyzed. Therefore, we expanded the search scope and updated our meta-analysis based on all published RCTs to evaluate whether this technology can reduce the incidence of AL in patients undergoing rectal cancer resection.

## Materials and methods

This systematic review and meta-analysis was conducted according to the Preferred Reporting Items for Systematic Reviews and Meta-analyses reporting guidelines (22). Accordingly, this study did not require ethics approval or informed consent.

### Study strategy

A systematic literature search was conducted to retrieve relevant data from PubMed, Embase, and Cochrane Library databases from inception until November 1, 2022. The following search terms were used: ((transanal catheter) OR (transanal drainage tube) OR (transanal tube) OR (rectal tube) OR (transanal)) AND ((Rectal) OR (Rectum)) AND ((Anastomotic Leak) OR (Anastomotic Leakage)). Additionally, the reference lists in these studies were evaluated to include more comprehensive studies.

Two authors (LJ-M and LD-L) independently reviewed the titles and abstracts of all retrieved studies and excluded the studies that did not meet the inclusion criteria. Subsequently, they conducted a full-text review of the selected studies to further determine the studies that met the criteria.

### Inclusion and exclusion criteria

The inclusion criteria were as follows: (a) studies involving patients undergoing anterior resection for rectal cancer, (b) studies comparing the incidence of AL in patients undergoing resection with and without TDT, and (c) RCTs.

The exclusion criteria were as follows: (a) non-RCTs, (b) TDT was used to treat patients with AL, (c) no TDT was used in the study, and (d) insufficient data.

### Data extraction

Data were independently extracted by two authors (LJ-M and LC-Y). In case of disputes and differences, a third author (Y-L) was consulted to reach a consensus. The extracted information included the name of the first author, year of publication, country of study, design of study, type of surgery, type of tube, depth of TDT placement, duration of TDT, number of stomas, number of AL cases, and classification of AL.

### Outcomes

This study primarily aimed to compare the incidence of AL in patients who underwent anterior resection for rectal cancer with and without TDT. The secondary subgroup analysis included differences in the incidence of AL among patients with diverting stomas and different grades.

### Statistical analysis

Review Manager, version 5.3 (Nordic Cochrane Center, Cochrane Collaboration, London, UK) was used for data analysis. The risk ratio (RR) was used as an effect measurement at 95% confidence interval (CI). Heterogeneity was described using I^2^ value and was divided into four levels: no heterogeneity (I^2^ < 25%), low heterogeneity (25% ≤ I^2^ < 50%), medium heterogeneity (50% ≤ I^2^ < 75%), and high heterogeneity (I^2^ ≥ 75%). The fixed model effect was used when the I^2^ value was <50%, whereas the random model effect was used when it was >50%.

## Results

### Selected studies

**Figure 1:**
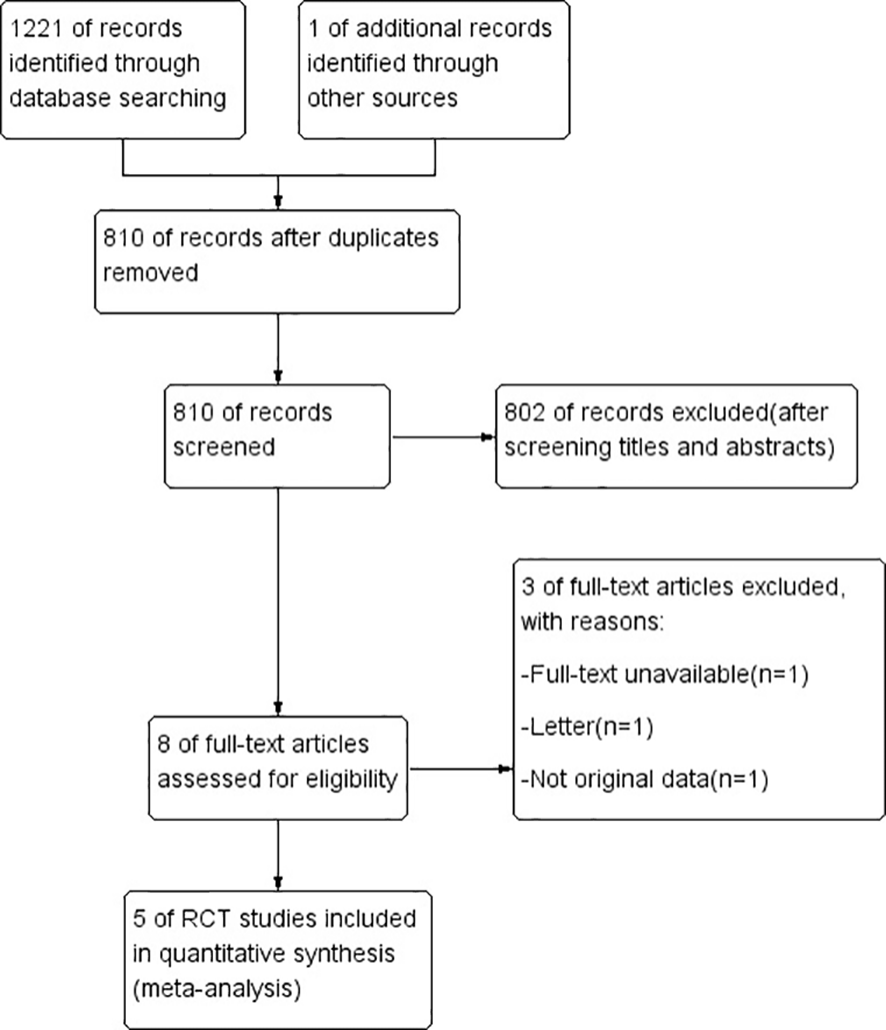
PRISMA flow diagram of study selection.

In total, 1222 studies were retrieved, of which 810 studies were evaluated after eliminating duplicates. Further, 801 studies were excluded because they did not meet the inclusion criteria, and only 5 (19, 20, 23–25) of the remaining 8 potential studies were finally included in this study. The detailed process of literature retrieval and screening is shown in **Figure 1**. These 5 studies were published between 2003 and 2021 and comprised 1385 patients from 4 countries (sample size, 76–580; **Tables 1**, **2**).

**Table 1:** Characteristics of the included trials.

Study	Year	Country	Study design	Type of surgery	Type of tube	Depth of TDT placement	Duration of TDT
Amin et al.(23)	2003	UK	RCT	LAR	silicone tube	Unmentioned	5–7 days
Bülow et al.(24)	2006	Denmark	RCT	AR	silicone stent	Unmentioned	4 days
Tamura et al.(25)	2021	Japan	RCT	LAR	Malecot latex tube	3-5cm	at least 5 days
Xiao et al.(19)	2011	China	RCT	LAR	silicone tube	Unmentioned	5–7 days
Zhao et al.(20)	2021	China	RCT	LAR	silicone tube	5cm	3–7 days

RCT, randomized controlled trial; AR, anterior resection; LAR, low anterior resection.

**Table 2:** Characteristics of the included trials.

Study	Sample size		Cases of AL		Grade A of AL		Grade B of AL		Grade C of AL		Cases of Ileostomy		Ileostomy of AL	
	TDT	Non-TDT	TDT	Non-TDT	TDT	Non-TDT	TDT	Non-TDT	TDT	Non-TDT	TDT	Non-TDT	TDT	Non-TDT
Amin et al.(23)	41	35	3	2	*	*	*	*	*	*	*	*	*	*
Bülow et al.(24)	98	96	17	8	*	*	*	*	*	*	44	45	9	3
Tamura et al.(25)	79	78	8	11	2	3	5	7	1	1	*	*	*	*
Xiao et al.(19)	200	198	8	19	*	*	6	3	2	16	*	*	*	*
Zhao et al.(20)	280	280	18	19	*	*	14	11	4	8	72	89	6	4

* means that data cannot be obtained.

### Quality assessment

**Figure 2:**
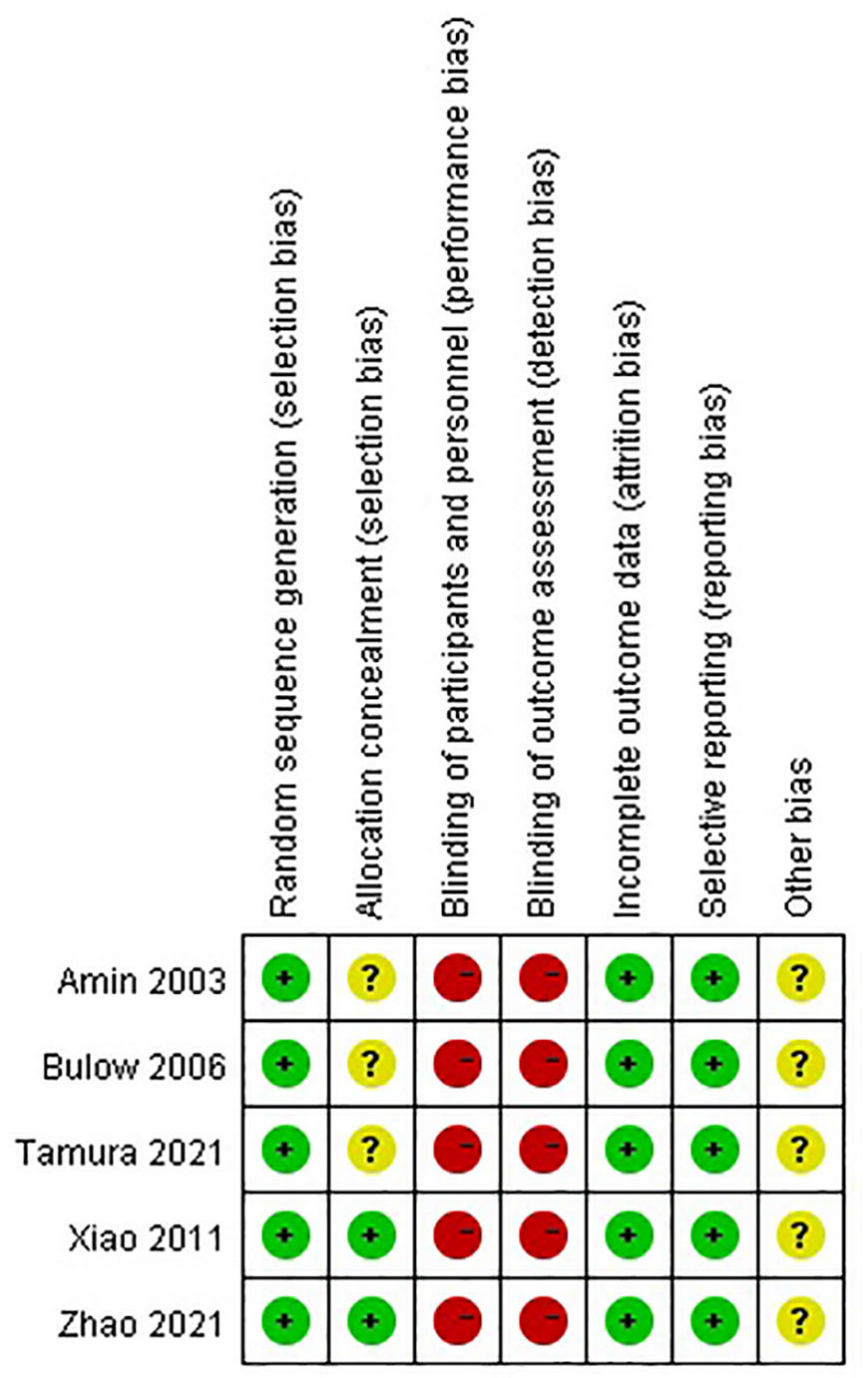
Summary of the risk of bias.

**Figure 3:**
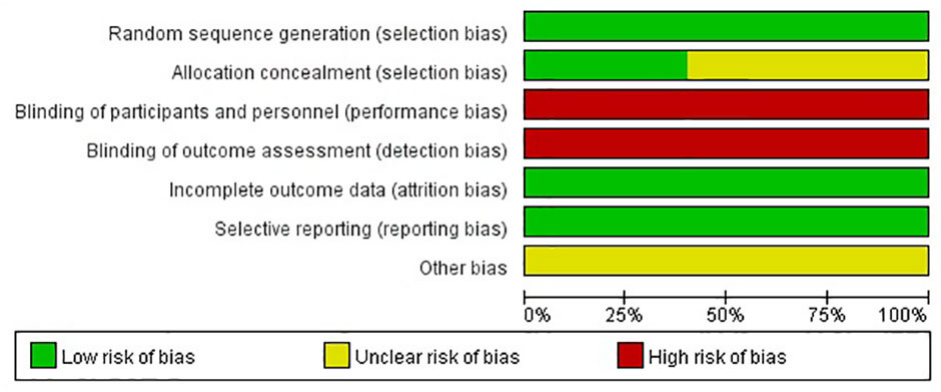
Graph of the risk of bias.

The results of the Cochrane risk of bias tool are shown in **Figures 2**, **3**. Considering the nature of intervention, it was difficult to conduct a blinded analysis with the researchers and participants.

### Outcomes

**Figure 4:**
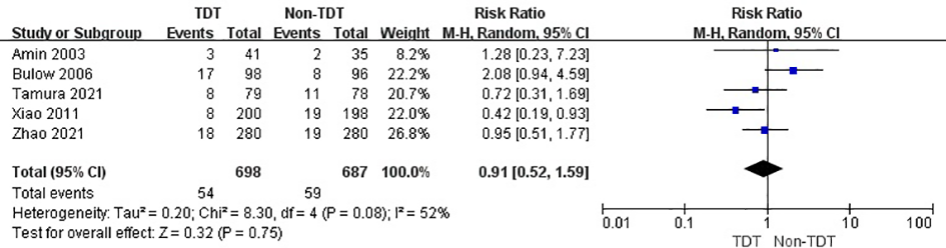
Overall incidence of AL in TDT and non-TDT groups.

Analysis of the five RCTs revealed that the overall incidence of AL in TDT and non-TDT groups was 7.7% and 8.6%, respectively (RR, 0.91; 95% CI, 0.52–1.59; p = 0.75; **Figure 4**), indicating that TDT did not reduce the incidence of AL.

**Figure 5:**
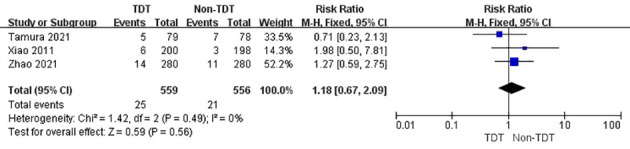
Incidence of grade B AL in TDT and non-TDT groups.

**Figure 6:**
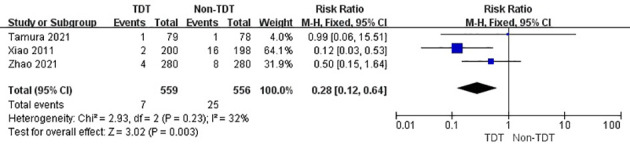
Incidence of grade C AL in TDT and non-TDT groups.

The summary analysis revealed a similar result in patients with grade B AL but not in those with grade C AL. The incidence of grade B AL in TDT and non-TDT groups was 4.5% and 3.8%, respectively (RR, 1.18; 95% CI, 0.67–2.09; p = 0.56; **Figure 5**), whereas that of grade C AL was 1.3% and 4.5%, respectively (RR, 0.28; 95% CI, 0.12–0.64; p = 0.003; **Figure 6**).

**Figure 7:**
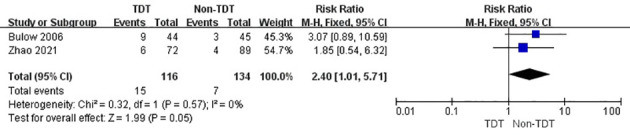
Incidence of AL in patients in TDT and non-TDT groups with a stoma.

Two RCTs indicated that AL occurred in patients with a stoma after rectal cancer surgery with and without TDT. The incidence of AL in TDT and non-TDT groups was 12.9% and 5.2%, respectively (RR, 2.40; 95% CI, 1.01–5.71; p = 0.05; **Figure 7**).

### Publication bias

In accordance with the criteria in the Cochrane Handbook for systematic reviews of interventions, publication bias was not analyzed because none of the groups comprised >10 studies.

## Discussion

Surgery is the cornerstone of curative treatment for rectal cancer (26). However, AL is a severe postoperative complication, regardless of the surgical method performed under laparotomy or laparoscopy (3). AL leads to prolonged hospitalization, increased hospitalization costs, and high mortality rates; moreover, 25%–37% of postoperative deaths in patients with colorectal cancer are related to AL (27). In addition, AL leads to a high risk of local recurrence and poor prognosis of rectal cancer after surgery (28).

Studies have reported that the incidence of AL ranges from 5% to 19% (3) [mean incidence of approximately 10% (29)]. This difference in incidence is related to several factors, such as regional differences, research inclusion criteria, and diagnostic criteria for AL. The diagnostic criteria for AL reported by most studies (30–32) are as follows: (1) feces and gas outflow from the abdominal drainage tube; (2) pus outflow from the abdominal drainage tube; (3) postoperative symptoms of fever and abdominal pain, signs of local or total abdominal peritonitis, and high number of inflammatory response indicators; (4) rectal finger palpation of AL or sigmoidoscopy revealing a fistula; (5) pus outflow through the anus or feces, blood, or pus outflow through the vagina; (6) computed tomography or magnetic resonance imaging findings of gas and fluid accumulation in the pelvic cavity, or overflowing of the contrast agent during contrast examination; and (7) confirmation of AL *via* surgery. The international rectal cancer research team, represented by Rahbari (30), classified AL after anterior resection for rectal cancer into three groups (grades A, B, and C) based on the need for active treatment intervention or surgical treatment. Grade A refers to AL without special intervention, grade B AL requires special intervention but not surgery, and grade C AL requires surgical treatment.

The high-risk factors for AL mainly include male sex, preoperative chemotherapy and radiotherapy, diabetes, obesity, long-term steroid use, surgical area contamination, distance between the anastomotic orifice and anal margin, late tumor stage, microcirculatory disorders, prolonged operation time, and intraoperative bleeding. In addition, AL is related to various surgical techniques and perioperative conditions (33): (1) poor anastomotic blood supply (injury of the vascular arch of the proximal bowel during surgery, or free distal mesorectum resulting in several bare areas in the rectal stump); (2) excessive anastomotic tension (the remaining colon is extremely short or not free enough after resection of the diseased intestinal tube, leading to an increase in anastomotic tension); (3) inappropriate operation of the stapler (the anastomotic quality is reduced or the anastomosis is torn due to excessive squeezing and unstable firing during surgery); (4) poor preoperative intestinal cleanliness and premature postoperative defecation, leading to high anastomotic pressure; and (5) rough pelvic drainage after surgery leading to pelvic effusion and infection, thereby affecting the healing of the anastomotic stoma.

With regard to various risk factors, colorectal surgeons have been committed to reducing the incidence of AL after surgery and constantly improving the surgical techniques and equipment. A meta-analysis showed that low ligation of the inferior mesenteric artery during radical resection of rectal cancer seemed to be associated with a lower risk of anastomotic leakage and overall morbidity (34). At the same time, “Bespoke “ robotic surgery maintains optimal blood flow by preserving blood vessels, thereby reducing the incidence of anastomotic leakage after left hemicolectomy (35). An effective intervention measure that can reduce the incidence will be highly beneficial in clinical practice. Preventive colostomy of the ileum or proximal colon is the most common prevention and treatment method, which can reduce the serious complications caused by this condition. However, a preventive stoma is associated with additional complications, which require a reoperation. This could increase the discomfort as well as psychological and economic burden of patients after surgery.

High intraluminal pressure is a risk factor for anastomotic dehiscence after rectal cancer surgery (31). TDT are useful for relieving the pressure in the lumen *via* the continuous excretion of feces and gas from the proximal large intestine along with continuous anal expansion; additionally, they reduce the contamination caused by feces flowing through the anastomosis. In 1997, Klein et al. (36) proposed that the use of TDT through the anus within 1–5 days after surgery can increase the safety of the anastomotic stoma. Subsequently, other studies (15, 32, 37) have evaluated the role of TDT placement in preventing AL and constantly improved the material and efficacy of TDT. However, the prevention and treatment of AL by the TDT remains controversial.

The five RCTs included in this meta-analysis reported different conclusions. Amin et al. (23) reported that the incidence of AL was similar in the two groups, regardless of the use of a transanal stent; however, clinical leakage requiring surgical intervention was more commonly observed when the stent was used in patients with AL. In a recent multicenter RCT, Zhao et al. (20) concluded that TDT did not play a role in preventing AL. This was consistent with the study by Bülow et al. (24)and Tamura et al. (25); however, in the study by Xiao et al. (19), TDT were effective and safe in reducing the incidence of symptomatic AL.

The current meta-analysis is the largest study comprising RCTs (5 articles and 1385 patients) on the effect of TDT on AL in patients who underwent rectal cancer surgery. The summary analysis of these patients revealed that the use of TDT during surgery did not reduce the incidence of AL after rectal cancer resection. Furthermore, TDT did not reduce the incidence of grade B AL but decreased the incidence of grade C AL after rectal cancer resection. TDT reduced the incidence of grade C AL by decreasing the extent of contamination caused by feces flowing through the anastomotic stoma and alleviating abdominal complications; therefore, the AL requires special intervention but no surgical treatment. The incidence of AL with and without TDT was significantly different among patients who had a preventive stoma after rectal cancer resection. Notably, the incidence of AL in patients who underwent resection without TDT was low, which may be attributed to few studies included in this analysis, leading to biased conclusions.

This study has some limitations. First, there were differences in the selection of the control group in the included RCTs. Some studies compared patients who did not use TDT, whereas others compared patients with preventive stomas, which may have potential publication bias. Second, the material, service time, and placement depth of TDT in these studies were not consistent. Finally, owing to the small sample size (five RCTs included in this study), it was difficult to construct a funnel chart. These limitations may lead to heterogeneity in the analysis of the results. Therefore, a more carefully designed RCT is warranted to clearly evaluate whether TDT can be used instead of diverting stoma to reduce the incidence of AL in patients undergoing anterior resection for rectal cancer.

## Conclusion

Using TDT during rectal cancer surgery is not associated with a significant reduction in AL. However, considering the abovementioned limitations, additional multicenter RCTs with larger sample sizes based on unified control standards and TDT indications are warranted to validate these findings.

## Author contributions

First author: SX (analysis and interpretation of data, drafting the article or revising it critically for important intellectual content). The corresponding author: WW (conception and design, final approval of the version to be published). Other authors: LM, LL, LY and YL (acquisition of data). All authors contributed to the article and approved the submitted version.
